# Medical Resources

**Published:** 1868

**Authors:** J. G. Westmoreland

**Affiliations:** Professor of Materia Medica in Atlanta Medical College, Atlanta, Georgia


### ARTICLE II.

MEDICAL RESOURCES. By J. G. WESTMORELAND, M. D., Professor of Materia Medica in Atlanta Medical College, Atlanta, Georgia.

In order to the successful treatment of disease, means adapted to the various forms in which it appears and the varied conditions of its subjects, must be always at command. There is a simple and common, but nevertheless wise, adage which runs thus: "Prepare for war in time of peace." That commander who neglects the equipment and arrangement of troops until the enemy has made the attack, is not less likely to succeed in routing him than the physician who undertakes to protect the system from the inroads of disease, without having at his command the means by which it is to be done, and a knowledge of the proper manner of using them. Every one knows that opium is an anodyne, and ordinarily quiets restlessness and pain, but this is not always the result of its use when these difficulties exist. Other articles are useful for the same purpose, and peculiarities are possessed by each, differing from the others, which make one more appropriate than another in certain contingencies. All the remedies known to the profession should, therefore, be studied in connection with the classes to which they belong. Not only so, but, as slight variations are found in the same disease, in the constitutional conformation of the patient, and in the circumstances in other respects surrounding different cases, the peculiar and varied minor properties of remedies should also receive due consideration, in order to judicious selection. The indications in the treatment of disease, the classes of remedies, and their *modus operandi*, which are to be wielded in filling those indications, are subjects for grave consideration, and require the exercise of profound judgment. If plans of treatment, one after another, fail, others, founded on rational views of the nature and treatment of disease, should be easily called to mind; and if article after article, of a particular class, fail to come up to the full measure of effect desired, still others should be familiarly known, out of which to select one more appropriate. These subjects of study make up the physician's resources. He that ignominiously abandons the wretched sufferer, after a puny effort to stay the disease with which he is afflicted, without such supply of varied resources, has not made that preparation necessary for so important responsibility. That physician most limited in these respects comes nearest the unenviable position of those who are content to trust all to expectancy or one-ideal systems of practice.

When Dr. Chapman reached the acme of his medical career, and was considered the most popular and successful practitioner in the United States, it was said of him, that "his resources were unbounded." Thus it is, that those who lay broad the foundation and keep pace with the discoveries of the age, do, by the direction of a sound judgment, prove a blessing to the world and an honor to the profession.

The science of medicine is progressive, and continually the discovery of new remedies, and new properties to medicines already in use, is being made. The catalogue and classification of remedies made twenty years ago would exhibit a very imperfect state of the science now. Whilst it is advancing in the accumulation of additional truths, its devotees must also be on the alert to keep pace with developments constantly being made. The number and medicinal properties of agents, as understood even in the days of Rush, would not afford resources sufficient to sustain respectability in a physician now. While we cannot penetrate the deep vista of the future sufficiently to realize even the possibility of reaching the goal of perfection, yet the cause of science and humanity urges to progression! progression!!

